# Relationship between somatic mosaicism of *Pax6* mutation and variable developmental eye abnormalities—an analysis of CRISPR genome-edited mouse embryos

**DOI:** 10.1038/s41598-017-00088-w

**Published:** 2017-03-03

**Authors:** Akihiro Yasue, Hitomi Kono, Munenori Habuta, Tetsuya Bando, Keita Sato, Junji Inoue, Seiichi Oyadomari, Sumihare Noji, Eiji Tanaka, Hideyo Ohuchi

**Affiliations:** 1 Department of Orthodontics Dentofacial Orthopedics, Institute of Biomedical Sciences, Tokushima University Graduate School, 3-18-15 Kuramoto-cho, Tokushima, 770-8504 Japan; 2 Department of Cytology and Histology, Okayama University Graduate School of Medicine, Dentistry and Pharmaceutical Sciences, 2-5-1 Shikata-cho, Kita-ku, Okayama 700-8558 Japan; 3 Division of Molecular Biology, Institute for Advanced Enzyme Research, Tokushima University, 3-18-15 Kuramoto-cho, Tokushima, 770-8503 Japan; 4 Tokushima University, 2-24 Shinkura-cho, Tokushima, 770-8501 Japan

## Abstract

The clustered regularly interspaced short palindromic repeat (CRISPR)/CRISPR-associated protein (Cas) system is a rapid gene-targeting technology that does not require embryonic stem cells. To demonstrate dosage effects of the *Pax6* gene on eye formation, we generated *Pax6*-deficient mice with the CRISPR/Cas system. Eyes of founder embryos at embryonic day (E) 16.5 were examined and categorized according to macroscopic phenotype as class 1 (small eye with distinct pigmentation), class 2 (pigmentation without eye globes), or class 3 (no pigmentation and no eyes). Histologically, class 1 eyes were abnormally small in size with lens still attached to the cornea at E16.5. Class 2 eyes had no lens and distorted convoluted retinas. Class 3 eyes had only rudimentary optic vesicle-like tissues or histological anophthalmia. Genotyping of neck tissue cells from the founder embryos revealed somatic mosaicism and allelic complexity for *Pax6*. Relationships between eye phenotype and genotype were developed. The present results demonstrated that development of the lens from the surface ectoderm requires a higher gene dose of *Pax6* than development of the retina from the optic vesicle. We further anticipate that mice with somatic mosaicism in a targeted gene generated by CRISPR/Cas-mediated genome editing will give some insights for understanding the complexity in human congenital diseases that occur in mosaic form.

## Introduction

Pax6 is a homeodomain-containing transcription factor that is critical for eye and nasal development in most animals, including *Drosophila*, mice, and humans, as well as in the neural development and differentiation of pancreatic endocrine cells^1–3^. It has been shown that the effects of *Pax6* mutation depend on extent of mutation and gene dosage. That is, large deletion mutation produces a more severe phenotype than hypomorphic mutations such as missense substitution of an invariant amino acid, and homozygous mutants are more severely affected than heterozygous mutants, which exhibit haploinsufficiency. For example, homozygous *Pax6*-null mutation in mice results in a failure to develop eyes and nasal cavities and embryonic lethality; meanwhile heterozygous *Pax6* mutation in humans results in small eyes and aniridia^4–6^.

The CRISPR/Cas system of RNA-guided genome editing is used as a facile and rapid gene targeting technique for altering genes in a wide variety of cell types, beyond embryonic stem cells, and in various organisms including mice^7, 8^. Cas9 nuclease, which is guided by single guide RNA (sgRNA), hybridizes specifically with and induces double-stranded breaks (DSBs) in complementary genomic sequences. The DSBs are repaired by either non-homologous end-joining (NHEJ) or homology-directed repair in the presence of donor DNA. Because NHEJ leads to small insertions and deletions, the open reading frame is disrupted, thereby inactivating the target gene.

Previously, we used the CRISPR/Cas system to generate *Fgf10*-knockout mice and obtained limbless mice, identical to those produced by conventional gene targeting techniques^9, 10^. In the genome-edited founder mice, varying degrees of mosaicism in mutations of targeted genes have been reported^11–13^. Here, we used the CRISPR/Cas system to generate *Pax6*-deficient mice. We focused on eye phenotype and examined relationship between genotype and phenotype of *Pax6*-mutated mice. We studied the effects of *Pax6* gene dosage on eye development by utilizing observation of *Pax6*-mosaic founder mice.

## Results

### CRISPR/Cas-mediated gene knockout

**Figure 1:**
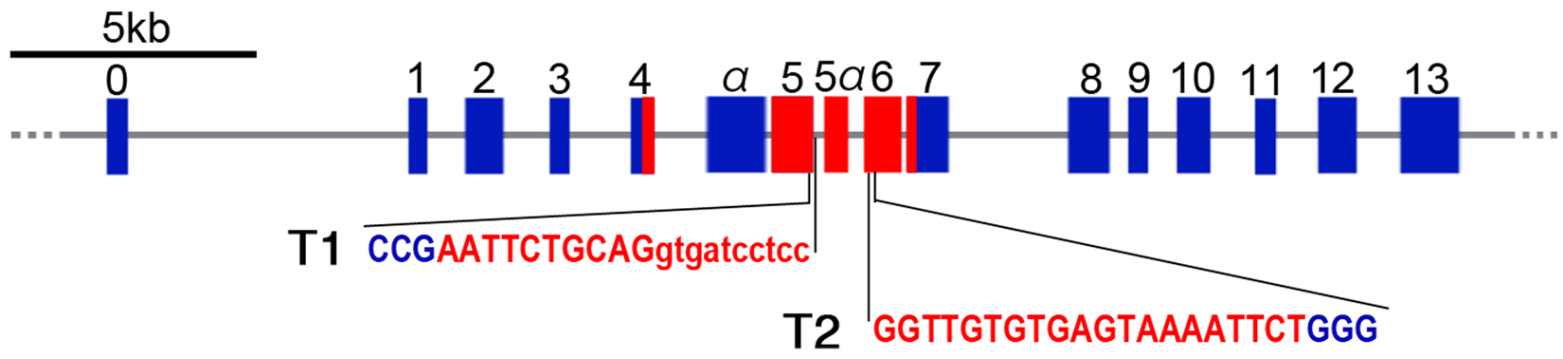
Mouse *Pax6* gene structure and single guide sgRNA design. Diagram of mouse *Pax6* locus with 16 exons. The red boxes show exons that give rise to the PD. The blue boxes show other exons. Sequences in exon 5 and exon 6 were targeted to generate two sgRNAs (T1 and T2, in red). PAM sequences are indicated in blue. Uppercase letters indicate coding sequence, and lowercase letters indicate intron sequence.

We designed target sites in mouse *Pax6* in exon 5 [target 1 (T1)] or exon 6 [target 2 (T2)], each of which encodes part of the paired domain (PD), as shown in Fig. 1. The PD was chosen for targeting because a considerable number of mouse *Pax6* mutants and human aniridia cases had mutations in the PD^14–16^. Furthermore, target sites were easily determined with respect to the proto-spacer adaptor motif (PAM; NGG nucleotides) sequence. T1 is located in the exon-intron boundary, whereas T2 is within exon 6. We microinjected T1 or T2 sgRNA and Cas9 mRNA into the cytoplasm of fertilized mouse eggs at the one-cell stage. After being cultured overnight, the injected zygotes were transferred into the oviducts of pseudo-pregnant females.

### *Pax6*-CRISPR founder mice exhibited varying degrees of eye defects

First, to know the gene knockout efficiency achieved by the CRISPR/Cas system, we examined the eye phenotypes of founder embryos at E16.5 under a dissecting microscope. The semidominant and homozygous lethal “*S* *mall* *ey* *e*” (*Sey/Sey*) mutation (a null mutation in *Pax6*) is known to cause failure of lens development from the head surface ectoderm, thereby aborting eye morphogenesis^3, 4^. Therefore, we anticipated that it would be easy to evaluate the efficiency of CRISPR/Cas system by observing the external morphology of founder embryonic eyes.

**Figure 2:**
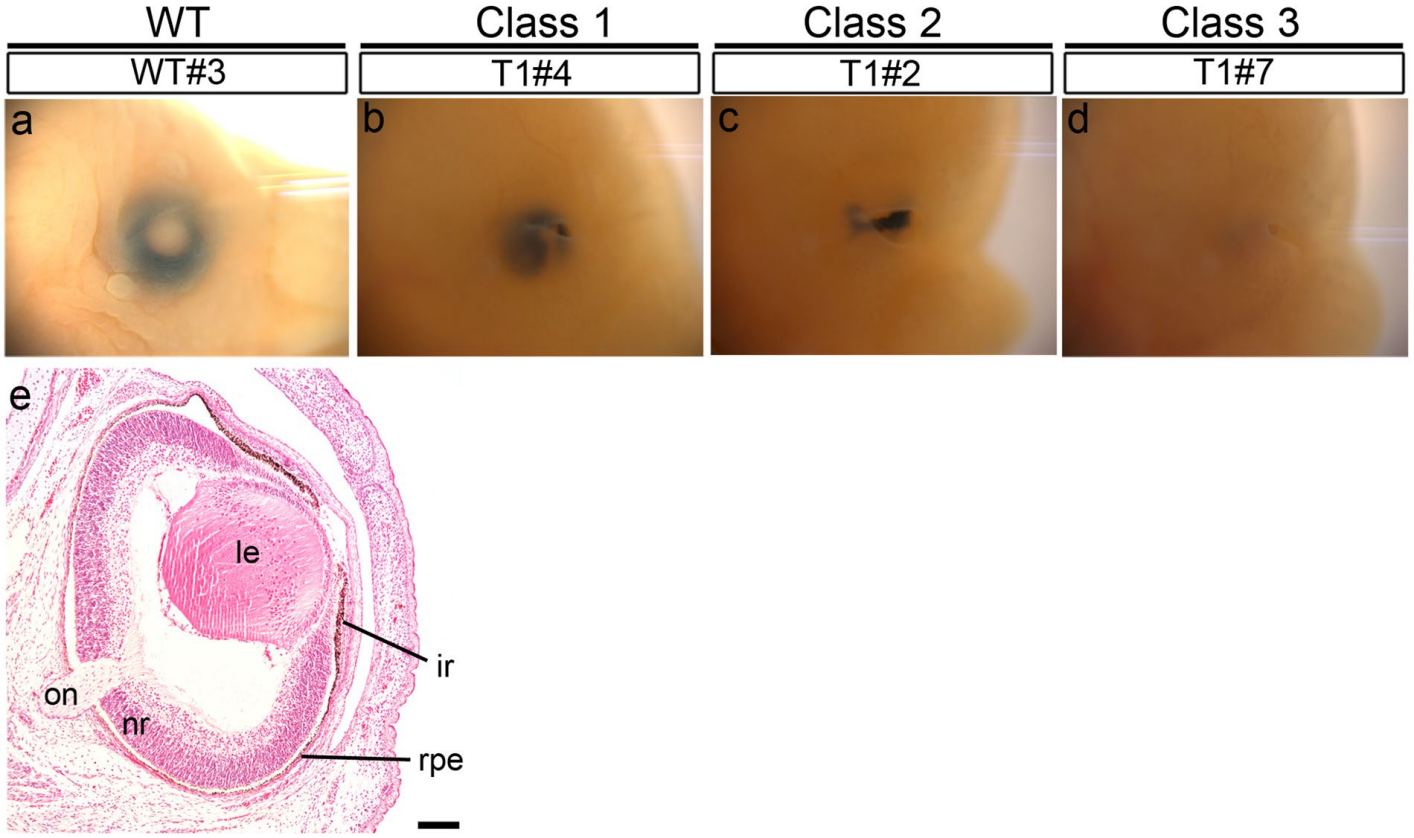
Eye phenotypes of *Pax6*-CRISPR founder mice at E16.5. (**a**–**d**) Lateral view of the eye on the right side. (**a**) Wild-type eye. Based on stereomicroscopic observations, we categorized the eyes into three phenotype classes: class 1 (**b**) class 2 (**c**) and class 3 (**d**). (**e**) HE-stained coronal sections of wild-type eye. The retina has differentiated to form the inner neuroblastic layer. ir, iris; le, lens; nr, neural retina; on, optic nerve; rpe, retinal pigmented epithelium. Scale bars: 100 *μ*m.

We observed embryonic eyes under a stereomicroscope and found varying degrees of eye malformation (Figs 2 and S1). We categorized the eyes into three phenotypes: class 1, small eye with distinct pigmentation (Fig. 2b; compare with Fig. 2a); class 2, pigmentation without obvious eye globes (Fig. 2c); and class 3, no pigmentation and no eyes (Fig. 2d). T1 embryos (N = 22) exhibited class 1 (48%, N = 12, 21 eyes), class 2 (32%, N = 10, 14 eyes), and class 3 (20%, N = 6, 9 eyes) phenotypes. Meanwhile, T2 embryos (N = 19) exhibited only class 2 (5%, N = 1, 2 eyes) and class 3 (95%, N = 18, 36 eyes) phenotypes (for details, see Table 1). Although larger numbers of severe eye phenotypes were observed in T2 than in T1 embryos, the samples are not large enough to support strong conclusions. Nonetheless, given this early pattern of phenotypes, it might be targeting within an exon, as in T2, disrupts genes more efficiently than altering an intron (Fig. 1). However, *in vitro* analysis of cleavage efficiency by Cas9 nuclease under the T1 or T2 sgRNA showed no obvious difference between these two target sites (Fig. S2). On the other hand, there were some embryos exhibiting size and morphological differences between right and left eyes; for example, T1#9 and T1#10 embryos have class 3 eyes on the right side and class 2 eyes on the left side (Fig. S3, Tables S1 and S2).

**Table 1:** Number of *Pax6*-CRISPR/Cas embryos examined by stereoscopy.

Phenotype	T1 (N = 22 : T1#1 ~ #22)		T2 (N = 19 : T2#1 ~ #19)	
	Right eye	Left eye	Right eye	Left eye
Class 1	11	10	0	0
Class 2	5	9	1	1
Class 3	6	3	18	18

### Correlation between external eye morphology and histology

**Figure 3:**
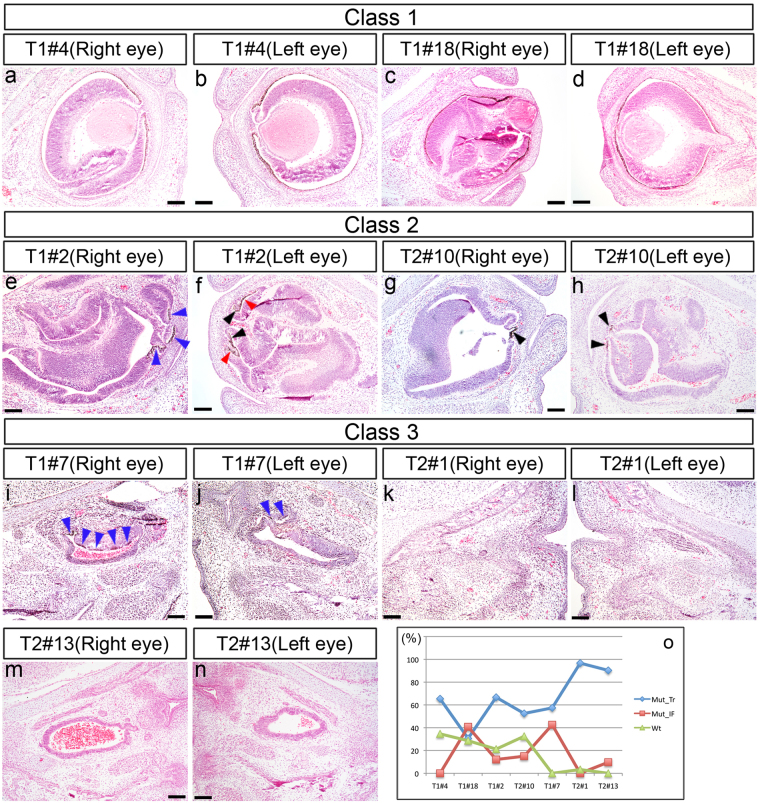
HE-stained eye tissues from founder mice at E16.5. Coronal sections are shown. (**a**–**d**) Class 1 eyes. (**e**–**h**) Class 2 eyes. (**i**–**n**) Class 3 eyes. In panels (**e**–**j**) the red arrowhead shows the prospective RPE, the blue arrowhead shows pigmented epithelial cells of the iris or RPE, and the black arrowhead shows the prospective iris. (**o**) Percentage of mutated and wild-type genotypes found in each embryo. Mut-Tr, mutations encoding truncated Pax6 proteins; Mut-IF, in-frame mutations; Wt, wild-type. Detailed results are shown in Fig. S5 and Table S3. Scale bars: 100 *μ*m.

To substantiate the stereoscopic eye phenotype, hematoxylin-eosin (HE)-stained coronal sections were examined histologically (28 eyes; N = 9 for T1, N = 5 for T2; Table 2). Representative results are shown in Figs 3 and S4. In both eyes of the normal embryonic day (E) 16.5 embryo, a lens and neural retina (NR) had formed with an optic nerve, retinal pigmented epithelium (RPE), and iris (Figs 2e and S4a,b). In the class 1 T1#4 embryo, both eyes were small and the lenses remained attached to the corneas (Figs 3a,b and S4c,d), which is characteristic to the *Pax6*-heterozygous eye^17, 18^. Furthermore, the right eye had a convoluted NR (Fig. 3a). In the class 1 T1#18 embryo, the right eye had a smaller lens and several separated NR-folds (Fig. 3c). In the class 2 T1#2 and T2#10 embryos, the eyes had no lenses and the retinas were distorted with multiple convolutions (Fig. 3e–h), a phenotype resembling that in embryos in which *Pax6* was specifically inactivated in the eye surface ectoderm^19^. The putative RPE cells were discernible, but appeared mostly with hypopigmentation and with columnar rather than mature cuboidal shaped cells (Fig. 3e,f). The putative iris cells appeared as patches of cells with pigmentation near the presumptive cornea (Fig. 3g,h). In class 3 embryos, there were no lenses whatsoever. For example, in the T1#7 embryo, vestigial optic vesicle-like epithelial tissues with pigmentation were observed (Fig. 3i,j). In the T2#1 embryo, which exhibited histological anophthalmia, there were no apparent optic vesicle-like tissues (Fig. 3k,l). In the T2#13 embryo, there were only vestigial optic vesicle-like epithelial tissues, but a complete absence of pigmented cells (Fig. 3m,n). Thus, the class 3 phenotype resembled the *Pax6*-null mouse phenotype^20^.

**Table 2:** Eye phenotypes of *Pax6*-CRISPR/Cas embryos examined histologically.

			Class	Lens	Iris	RPE	NR
**T1**	**#4**	**R**	**1**	N	N	h	h
		**L**	**1**	N	N	N	N
	**#6**	**R**	**1**	N	N	N	N
		**L**	**1**	N	N	N	N
	**#18**	**R**	**1**	N	N	h	h
		**L**	**1**	N	N	N	N
	**#2**	**R**	**2**	a	h	h	h
		**L**	**2**	a	N	h	h
	**#9**	**L**	**2**	a	h	h	h
	**#12**	**R**	**2**	a	h	h	h
		**L**	**2**	N	N	h	h
	**#13**	**L**	**2**	a	h	h	h
	**#7**	**R**	**3**	a	h	h	h
		**L**	**3**	a	h	h	h
	**#9**	**R**	**3**	a	h	h	h
	**#11**	**R**	**3**	a	h	a	h
		**L**	**3**	a	h	h	h
	**#13**	**R**	**3**	a	h	h	h
**T2**	**#10**	**R**	**2**	a	h	a	h
		**L**	**2**	a	n	a	h
	**#1**	**R**	**3**	a	a	a	a
		**L**	**3**	a	a	a	a
	**#3**	**R**	**3**	a	a	a	h
		**L**	**3**	a	a	a	a
	**#8**	**R**	**3**	a	a	a	h
		**L**	**3**	a	h	h	h
	**#13**	**R**	**3**	a	a	a	h
		**L**	**3**	a	a	a	h

**Figure 4:**
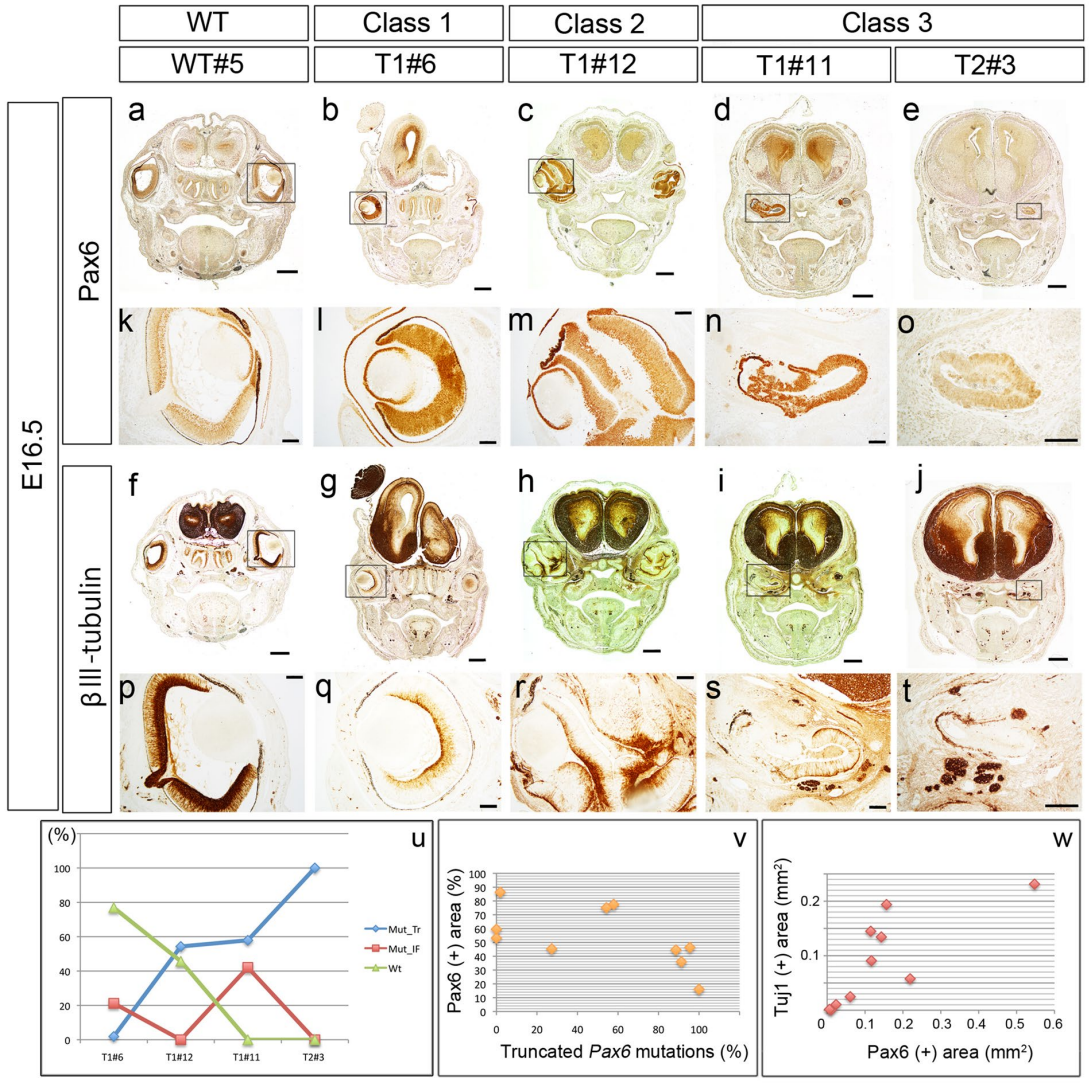
Pax6 and Tuj1 immunoreactivity. Localization of Pax6 (**a**–**e**,**k**–**o**), and *β*III tubulin (**f**–**j**,**p**–**t**) proteins in wild-type (left 4 panels) and *Pax6*-CRISPR (rest of the panels) embryos at E16.5. High magnification images of eyes (boxed area in a–j) are shown in (**k**–**t**). Serial coronal sections were immunolabeled with anti-Pax6 and anti-*β*III tubulin (Tuj1) antibodies. (**a**,**k**) In the E16.5 normal retina, expression of *Pax6* is downregulated and restricted to retinal ganglion cells of the inner neuroblastic layer and to the innermost cells of the outer neuroblastic layer. (**b**,**g**) Oblique section from the T1#6 embryo confirming that both eyes were categorized appropriately as class 1 phenotype. (**f**,**p**) In the E16.5 normal retina, *β*III tubulin is intensely detected in the inner neuroblastic layer. (**l**,**q**) Retinal development has delayed and the retina is not layered yet. Pax6 protein is detected all over the retina (**l**). *β*III tubulin is restricted to the innermost thin domain where differentiating retinal ganglion cells reside (**q**). (**n**) The anti-Pax6 antibody detects abnormal Pax6 protein in which 3 amino acids are inserted in the PD. (**u**) Percentage of mutated and wild-type genotypes found in each embryo. Mut-Tr, mutations encoding truncated Pax6 proteins; Mut-IF, in-frame mutations; Wt, wild-type. Detailed results are shown in Fig. S5 and Table S4. (**v**) Relationship between the percent Pax6-positive retinal area and the percent truncated Pax6 mutations. The percent Pax6-positive retinal area showed an apparent negative correlation with the percent truncated *Pax6* mutations, but it was not statistically significant (r_s_ = −0.620; *P* = 0.056). (**w**) Similarly, the estimated area (mm^2^) of Tuj1-positive retina was positively correlated with that of Pax6-positive retina (r_s_ = 0.830; *P* = 0.003). Scale bars: 500 *μ*m in (**a**–**j**) and 100 *μ*m in (**k**–**t**).

T1, N = 9; T2, N = 5. The number after each # is identifies each embryo. R, right eye; L, left eye. N, normal structure; h, hypoplasia; a, agenesis. T1#6 eyes had normal histology, with delayed retinal development (see Fig. 4l,q) vs. wild-type (Fig. 4k,p).

The histologically observed abnormalities are summarized in Table 2. Only one (T1#6) out of 14 embryos (7%) examined had histologically normal eye structures on both sides; the rest had defects of some sort in at least one eye. However, even in T1#6’s eyes, retinal development appeared to be delayed (Fig. 4l,q; described later). Our histological observations support the notion that classification of *Pax6*-CRISPR eye phenotypes based on external morphology corresponds with the extent of histological abnormality fairly well.

### Genomic analysis of *Pax6*-CRISPR founder mice

To elucidate phenotype-genotype associations, we analyzed the genomic sequences of *Pax6*-targeted regions amplified by PCR. We analyzed genomic DNA samples from 20 founder embryos, including 15 of E16.5 embryos and 5 of E11.5 embryos: Each genomic PCR product was ligated to a plasmid DNA, the ligation products were transformed, and a set of resultant clones (22–89) was sequenced (Figs S5 and S6). We found targeted mutations (i.e. deletions and insertions) in the T1 or T2 regions along with wild-type sequences. For example, in T1#2, seven different mutations were found, including deletion of 12 nucleotides that likely abolished a splice donor site, resulting in a truncated gene product. 66.7% of sequenced clones showed the *Pax6* mutations resulting in the truncated proteins (Fig. S5 and Table S3). In T1#7, we observed a two-amino acid (Arg44-Ile45) deletion (42.6%), which, based on a human *PAX6* mutation database^16^, would be expected to result in translation of a Pax6 protein with reduced DNA-binding activity. Some of the founder embryos had frame-shift mutations resulting in the truncated proteins due to premature stop codons. For example, T1#18 carried three types of frame-shift mutations (30.6%) and one type in-frame mutation (40.8%) (Fig. S5 and Table S3). The in-frame mutation in T1#18 caused a three-amino acid (Arg-Ile-Leu) deletion in the PD. Although we did not determine the effects of particular types of *Pax6* mutations on specific eye phenotypes, we compared percentages of mutated sequences that result in C-terminal truncated Pax6 proteins (null function), in-frame mutations (null or reduced function), and wild-type sequences in each embryo histologically examined (Fig. 3o). We found that in class 3, the most severe eye phenotype, that histologically resembles those caused by null mutations, there is high incidence of truncated mutations and almost no wild-type Pax6 protein. These results suggest a correlation between the eye phenotype severity and the percent *Pax6* mutations revealed by sequencing and further highlight somatic mosaicism and allele complexity of the *Pax6* gene in founders generated by CRISPR/Cas-mediated mutagenesis of zygotes.

### Immunohistochemistry of Pax6 and *β*III tubulin

To reveal the localization of residual *Pax6*-expressing cells in the *Pax6*-CRISPR founder embryos, we performed immunohistochemistry on coronal head sections with an anti-Pax6 antibody (Fig. 4a–e,k–o). Because we used an anti-Pax6 antibody raised against the C-terminus of the mouse Pax6 protein (see Materials and Methods), Pax6-immunoreactivity indicated wild-type (+/+) or heterozygous (+/−) *Pax6*-expressing cells, and potentially also *Pax6-*homozygous mutated cells in which in-frame insertions and deletions retained normal C-terminal amino acid structures.

In the normal developing eye, *Pax6* is expressed in lens epithelial cells and retinal progenitor cells, and is expressed later in the anterior peripheral retina, retinal ganglion cell layer, and inner half of the inner nuclear layer^20–22^ (Fig. 4a,k). In the class 1 T1#6 eye, for example, which exhibited an overall normal structure, abundant Pax6 protein was present in the lens epithelium and in retinal progenitor cells of the NR (Fig. 4b,l). However, the NR was immature without a layered structure. In the class 2 T1#12 eye, the retina was distorted with multiple convolutions, possibly due to the hypoplastic lens (Fig. 4c)^19^. Pax6 protein was localized distinctly within the separated NR-folds (Fig. 4m). In the class 3 T1#11 embryo, a Pax6 protein was detected in the residual optic vesicle tissues (Fig. 4d,n). However, genomic analysis showed that the embryo had two types of *Pax6* mutations: One encodes a C-terminal truncated Pax6 (57.9%) and the other a 3 amino acid insertion in the PD (42.1%) (Fig. 4u and Table S4). This means that the anti-Pax6 antibody detected the mutated protein that is functionally defective and virtually null as the eye phenotype exhibits nearly anophthalmia. On the other hand, in the T2#3 embryo, the level of immunoreactivity was quite low (Fig. 4e,o), in which the mosaicism of *Pax6* mutations was found but all clones so far sequenced were mutated to encode C-terminal truncated proteins (Fig. 4u and Table S4). These observations suggest that residual *Pax6*-expressing cells (wild-type and/or *Pax6*-heterozygous) contribute to reduced or hyperproliferated retinal structures. However, these embryos obviously had not enough functional *Pax6*-expressing cells required for formation of wholly normal eye morphology.

We next performed immunohistochemistry for the neuron-specific marker *β*III tubulin (Tuj1) to assess whether the NRs of *Pax6*-CRISPR founder embryos initiated neural differentiation (Fig. 4f–j,p–t). Normal E16.5 retina displayed the characteristic pattern of neuronal cell differentiation, as indicated by Tuj1 immunopositivity in retinal ganglion cells, the first differentiating retinal cells^23^ (Fig. 4f,p). In class 1 T1#6 retina, a similar, but apparently developmentally earlier Tuj1 expression pattern was observed (Fig. 4g,q); that is, substantially fewer Tuj1-positive retinal ganglion cells were observed than in wild-type retina (Fig. 4f,p). In class 2 T1#12 eye tissue, the autonomous development of each retina fold was demonstrated by a normal appearing Tuj1 distribution within each of the NR folds (Fig. 4h,r). Localization of Tuj1 was observed even in the residual optic vesicle-like tissues of class 3 eyes (Fig. 4i,j,s,t), indicating that neuronal cell differentiation had at least started in the *Pax6*-CRISPR founder retina.

Previous studies using chimeras, trangenics, and analysis of *Pax6*-heterozygouts have shown that Pax6 works in a highly dose-dependent manner. Regarding this, we sought to examine whether the incidence of *Pax6*-mutations relates to the amount of functional Pax6 protein. We measured the area of Pax6-positive domains on immunostained sections and that of the entire retina. A scatter plot was built between percentage of Pax6-positive area per retina and that of truncated *Pax6* mutations (Fig. 4v). We found that a moderate negative correlation was observed between them, but it was not statistically significant (Fig. 4v). In contrast, a scatter plot between the Tuj1-positive area and that of Pax6-positive area showed a significant positive correlation between them (Fig. 4w). Thus, in this study, the area of Pax6-positive domains revealed by immunostaining almost reflects the presence of wild-type or C-terminal retained Pax6 proteins in *Pax6*-CRISPR founder retina. The positive correlation between the area of Pax6 and Tuj1 indicates that Pax6-gene dosage may have a positive effect on the expansion of Tuj1-posive cells, retinal ganglion cells.

### *Pax6*-mosaic optic vesicle is composed of Pax6-positive/-negative cells

**Figure 5:**
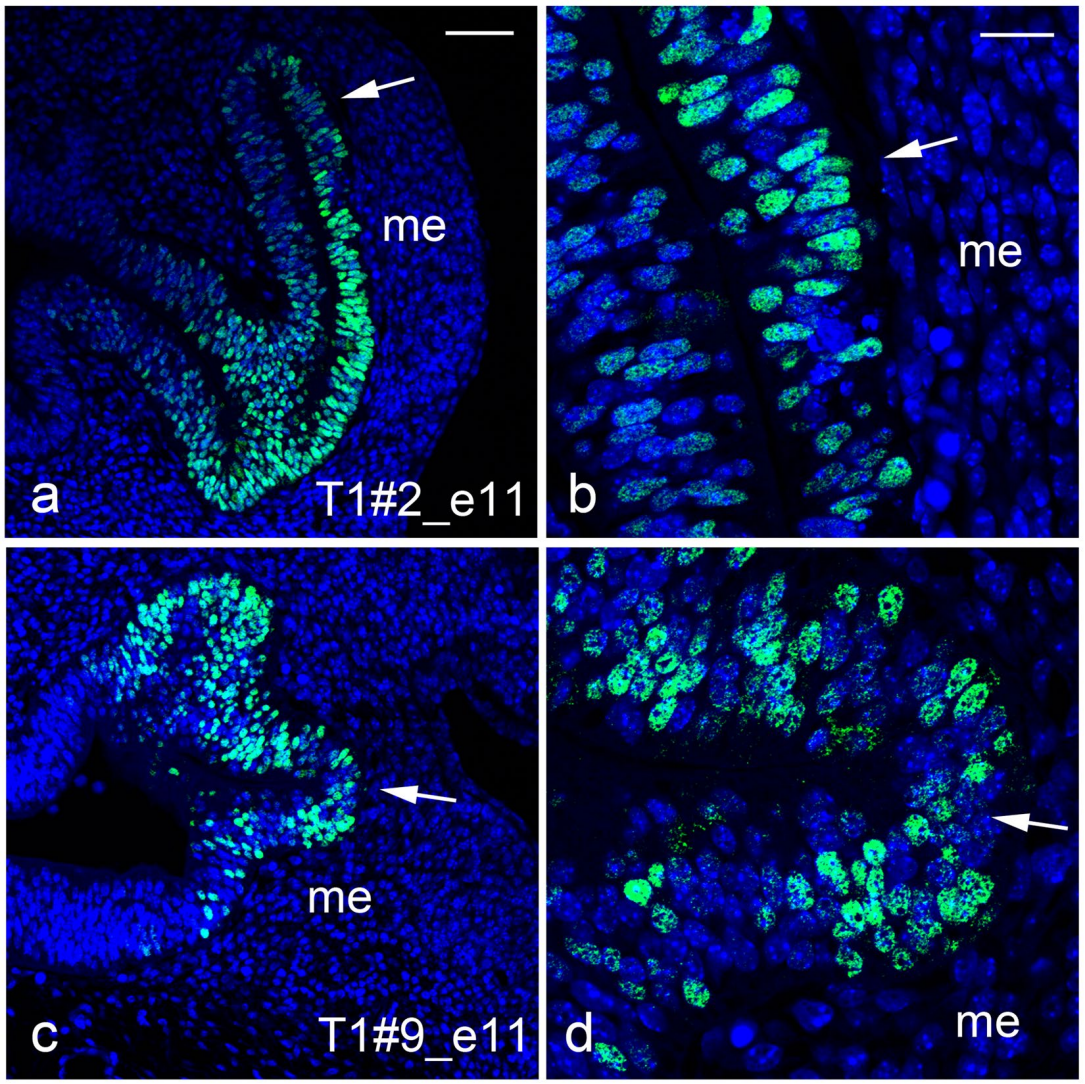
Pax6 immunoreactivity in the mosaic optic vesicle at E11.5. (**a**,**b**) Representative transverse sections of embryonic heads from T1#2_e11 and T1#9_e11, respectively. Green indicates localization of Pax6 protein and nuclei are stained with DAPI (blue). The arrow in (**a**,**c**) indicates the retinal neuroepithelium enlarged in (**b**,**d**), respectively. me, head mesenchyme surrounding the optic vesicle. Scale bars: 50 *μ*m in (**a**,**c**) and 20 *μ*m in (**b**,**d**).

To understand how the mosaic pattern of the retinal cells segregate, we examined eye phenotypes of *Pax6*-CRISPR founder embryos at E11.5. In normal embryos, the optic cup and lens vesicle form by E11.5. In some *Pax6*-CRISPR founder embryos, the neuroepithelium of optic vesicles overproliferated and there was no development toward the optic cup nor lens development (class 2) (Fig. S7a–d,q–t). In other founder embryos, in addition to the lack of lens vesicles or placodes, development of optic vesicles ceased (class 3) (Fig. S7e–h,m–p). Histological sections were immunostained with the anti-Pax6 antibody. We found that Pax6-positive cells and -negative cells were intermingled in the deteriorated optic vesicles (Fig. 5). In the T1#2 embryo (class 2), Pax6-postive cells were wild-type or heterozygous cells, whereas Pax6-negative cells were those with truncated (null) Pax6 proteins (Figs 5a,b and S6, S7u, Table S7). In the T1#9 embryo (class 3), Pax6-postive cells were those with defective Pax6 proteins (3 amino acid deleted in the PD) and Pax6-negative cells were those with truncated (null) Pax6 proteins (Figs 5c,d and S6, S7u, Table S7). In both embryos, it appears that Pax6-positive cells and -negative cells were segregating: There were portions where Pax6-negative cells are grouped together in the optic vesicle (Figs 5b,d and S8). Thus, around E11.5 the mosaic pattern of the cells with different genotypes is segregating in the *Pax6*-mosaic mouse.

## Discussion

In this study, morphological and genomic analyses demonstrated that our *Pax6*-CRISPR founder embryos with different *Pax6* mutations had eye phenotypes of varying degrees of hypoplasticity (class 1 to 3). Although how particular types of *Pax6* mutation determined yield specific phenotypes is not unknown in the present study, we sought to assign the mutants’ possible genotypes based on phenotype and affected genomic region sequence. Histology showed that the lenses in class 1 eyes (T1#4 and #18) had failed to detach from the cornea and the retina was folded. The *Pax6-*heterozygote (*Sey*/+) mouse has an abnormally small and vacuolated lens with folding of the optic cup margins^24^, and a diploid level of *Pax6* is required for detachment of the lens vesicle from the surface ectoderm^17^. Therefore, the genotypes of our class 1 embryos may be comparable to *Pax6*+/− heterozygosity or a mixture of wild-type (*Pax6*+/+) and bi-alleic *Pax6* mutations (*Pax6−*/−). The retinal development of the class 3 eyes had terminated at the optic vesicle stage or exhibited histological anophthalmia, similar to eye tissues found in *Pax6-*null mutants^20^. Histology of class 2 eyes showed no or reduced lens formation but convoluted retinas, which fits with a phenotype that is intermediate between class 1 and 3. This implies that intermediate level of *Pax6* expression may lead to retinal overproliferation per se, not due to lens deficiency.

Founder generations of frogs with *Pax6-*targeted genome editing^12, 25^ have been described as having mosaic eye phenotypes similar to those observed in this study. A recent paper shows that using electroporation, Cas9 protein with sgRNA, and early pronuclear zygotes is a most effective way to generate non-mosaic mutants in the mouse^13^. Our results indicated genetic mosaicism ranging from two to eight including wild-type (Tables S3, S4 and S7). This could be interpreted as that before E0.8 the translational machinery is not completely activated in the zygotes^13^, and therefore Cas9 is not effective at the time of microinjection, but nuclease activity of Cas9 might drag on as cleavage proceed. Thus, balancing of the concentration of Cas9/sgRNA injected must be useful to regulate genetic mosaicism, which awaits a further study.

So far, detailed analyses of eye phenotypes and their correlations with genotypes in F0 animals validating the influence of *Pax6* gene dosage on eye development have not been reported. Outcomes of altering Pax6 levels on eye development have been examined in studies of *Pax6-*heterozygous null mutants^17^, *Pax6* chimeras^26–28^, mice with conditional inactivation of *Pax6* ^19^, and *Pax6*-overexpressing transgenic mice^29^. Additionally, series of *Pax6-*hypomorph alleles have been used in gene-dosage studies. For example, the *Pax6* ^*7Neu*^ mutation, which is a base pair substitution in the Kozak sequence, results in a reduced level of Pax6 translation product^14^. In the resultant *Pax6-*hypomorph homozygous mutant, lenses do not develop, but rudimentary retinas do, as in the class 3 eyes in the present study (Fig. 3i,j). Taken together, analysis of our *Pax6*-mosaic founder mice leads to the conclusion that the surface ectoderm, which develops into the lens and cornea, is more sensitive to reduced levels of Pax6 than the optic vesicle, from which the retina and iris develop^3, 30^.

Although the importance of Pax6’s role in regulating proliferative rates depends on local expression of other regulatory factors, Pax6 is required invariably for proliferation of retinal progenitor cells and their multipotency^3^. Therefore, the rudimentary and immature retina with only initial differentiation (Tuj1 expression) observed in class 3 eyes may be explained by repression of proliferation due to Pax6 deficiency. Previous Pax6 chimera experiments showed that *Pax6*−/− and *Pax6*+/− cells segregate to form an ectopic optic vesicle or lens vesicles and postulated that it is due to different adhesive properties of wild-type and mutant cells^27^. Although we did not detect ectopic optic or lens vesicles in E11.5 *Pax6*-CRISPR founder embryos, segregation between the cells with different *Pax6*-genotypes also occurred in the abnormal optic vesicles, which supports the notion that Pax6 regulates a number of cell surface and adhesion molecules^31^.

Congenital aniridia (OMIM 106210) is a panocular disorder characterized by iris hypoplasia and reduced visual acuity^16, 32^. Classical isolated aniridia is a haploinsufficiency disorder caused by mutations in the *Pax6* gene. This anomaly is inherited with high penetrance, albeit with variable expressivity, and approximately a third of congenital aniridia cases are sporadic with no family history^33^. Variable expressivity of *Pax6* mutations has been explained by mosaicism, mutation type (chromosomal abnormality, nonsense mutation, missense mutation, etc.) and other factors apart from the *Pax6* mutation itself^15, 34^. Somatic mosaicism has been observed in other genetic diseases, such as retinitis pigmentosa and retinoblastoma, as well^35^. Many endogenous factors (e.g., mobile elements, DSBs in DNA, unbalanced chromosomal segregation) and some exogenous factors (e.g., nicotine and UV exposure) can generate somatic mutations, thereby causing somatic mosaicism^36^. Genome editing may be used to produce a mosaic mixture of mutant and wild-type cells in tissues. Thus, we envision that making and studying CRISPR/Cas-mediated mosaic mice with particular mutations of interest will help to elucidate why human congenital diseases exhibit variable expressivity and to reveal how somatic mosaicism caused by genetic and/or environmental factors is involved in human developmental abnormalities.

## Methods

### Animals

All animal care and experiments were carried out in accordance with the Guideline for Animal Experiments of Tokushima University and were approved by the Ethics Committee of Tokushima University for Animal Research (approval numbers 10110 and T27-16).

### Generation of *Pax6*-CRISPR mice

Two sites encoding a portion of the PD (T1, T2) were selected as target sites (Fig. 1). Potential off-target sites in exonic regions were searched using CRISPR Design (http://crispr.mit.edu)^37^ and listed as Table S8. Eye phenotypes we observed were not phenocopied by any knockouts of the candidate off-target genes listed. Production of *Cas9* mRNA and T1 or T2 sgRNA, and RNA microinjection into mouse zygotes were performed as previously described^9^. Microinjection experiments were performed twice for each target site.

### DNA sequencing of mutated endogenous gene target sites

Genomic DNA was isolated from the neck of fixed mouse embryos with a DNeasy Blood & Tissue Kit (Qiagen, Hilden, Germany). We used KOD-Plus-Neo (Toyobo, Osaka, Japan) or ExTaqHS (Takara, Kusatsu, Japan) and gene specific primers to isolate partial genomic DNA for Pax6_T1 and Pax6_T2 by PCR. The nucleotide sequences of the primers for Pax6_T1 were 5′-GAA TCA GCT TGG TGT CTT T-3′ and 5′-GGT GGT GTA GTC AGT AAT TAG CGT-3′, and those for Pax6_T2 were 5′-CTC GTA CCA TTG AAG GTA TAT TTT TGT-3′ and 5′-TTA TAC TGG GCT ATT TTG CTT ACA ACT-3′. The sizes of the desired amplicons were 253 bp for Pax6_T1 and 301 bp for Pax6_T2. The purified PCR amplicons were subcloned as described^9^ or into pGEM-T-Easy plasmids (Promega, Fitchburg, WI). After transformation, plasmids were extracted from the resultant bacterial colonies or colony PCR was performed. Sequencing was performed in our laboratory with an ABI 3500xL Genetic Analyzer (Applied Biosystems, Foster City, CA) as described previously^9^or by Eurofins Genomics (Tokyo, Japan). Mutated alleles were identified by comparison with the wild-type sequence in Genetyx-Mac software (Genetyx Co., Tokyo, Japan) and ClustalW 2.1 accessed via the DNA Data Bank of Japan.

### Histology and immunohistochemistry

Founder E16.5 and E11.5 embryos were obtained through Caesarian section and fixed in 4% paraformaldehyde in phosphate buffered saline overnight. After dehydration in serial ethanol series and clearing in xylene, the embryos were embedded in paraffin and cut into 5-*μ*m-thick sections for histology and 10-*μ*m-thick sections for immunohistochemistry. After deparaffinization, HE staining was performed according to standard procedures. Immunohistochemistry was performed by a Dextran polymer signal amplification system (EnVision HRP; Dako, Glostrup, Denmark). Anti-Pax6 (rabbit IgG, 1:1000 dilution; Covance, Princeton, NJ) and anti-***β***III tubulin (Tuj1; mouse IgG, 1:500 dilution; Covance) primary antibodies were used for immunolabeling. The anti-Pax6 antibody was generated against the peptide (QVPGSEPDMSQYWPRLQ) derived from the C-terminus of the mouse Pax6 protein. Immunoreactivity was visualized by a peroxidase reaction with 3,3′-diaminobenzidine. Micrograph images were taken with a Nikon digital camera (DS-Ri1; Nikon, Tokyo, Japan) and processed in Adobe Photoshop CS 5.1 (Adobe Systems Inc., San Jose, CA). Histological sections from E11.5 embryos were partly immunostained using fluorescent secondary antibodies (Alexa Fluor 488 conjugate, anti-rabbit IgG; Thermo Fisher Scientific) and DAPI, and their images were taken with a Zeiss confocal laser-scanning microscope (LSM 780; Zeiss, Jena, Germany).

### Morphometric analysis and statistics

The embryos containing all three classes were selected. In each embryo on each side, two serial (in principle) sections with the largest eye size were further selected and images of immunostained eyes were captured by a Nikon CCD camera. The area of Pax6-positive domains in the immunostained retinal sections was measured using ImageJ (https://imagej.nih.gov/ij/index.html). Tuj1-immunostained area and retinal domains were also measured in the same way. Briefly, “unit of length” was set as *μ*m and the images were changed to 8 bit. The weak but obviously stained portion in each image was marked using “freehand selections” and “enhance contrast” was processed under the following conditions: saturated pixels 5%, normalize, and update all when normalizing. Further, two types of filtering, “median” (radius 3.0 pixels, and 6.0 for larger magnification) and “unsharp mask” (sigma 3.0 or 6.0 pixels, mask weight 0.7) were processed. Threshold adjustment was set as “Otsu”. The retinal domains to be measured were marked by “freehand selections” tool and the domains to be analyzed were selected, converted to black, and the areas (*μ*m^2^) were measured. “The retina” here included neural retina, retinal pigmented epithelium, and optic nerve, and excluded lens, retinal vessels and blood cells. Statistical analysis of Chi-squared test and calculation of Spearman’s rank correlation coefficient was performed using IBM SPSS Statistics ver.24 (IBM Corporation, Armonk, NY).

## Electronic supplementary material

Supplementary Information 1

Supplementary Information 2
