# Supplementary material for: Relationship between somatic mosaicism of Pax6 mutation and variable developmental eye abnormalities—an analysis of CRISPR genome-edited mouse embryos
Source: Sci Rep. 2017 Mar 3;7:53. doi: 10.1038/s41598-017-00088-w (PMC5428340; doi:10.1038/s41598-017-00088-w)
Supplement: Supplementary file 1 — Supplementary Information 1 [file 41598_2017_88_MOESM1_ESM.pdf]

Supplementary Information 1

Relationship between somatic mosaicism of *Pax6* mutation and variable developmental eye abnormalities—an analysis of CRISPR genome-edited mouse embryos

Akihiro Yasue[1,*], Hitomi Kono[2], Munenori Habuta[2], Tetsuya Bando[2], Keita Sato[2], Junji Inoue[2], Seiichi Oyadomari[3], Sumihare Noji[4], Eiji Tanaka[1] and Hideyo Ohuchi[2,*]

[1]Department of Orthodontics Dentofacial Orthopedics, Institute of Biomedical Sciences, Tokushima University Graduate School, 3-18-15 Kuramoto-cho, Tokushima, 770-8504, Japan. [2]Department of Cytology and Histology, Okayama University Graduate School of Medicine, Dentistry and Pharmaceutical Sciences, 2-5-1 Shikata-cho, Kita-ku, Okayama, 700-8558, Japan. [3]Division of Molecular Biology, Institute for Advanced Enzyme Research, Tokushima University, 3-18-15 Kuramoto-cho, Tokushima, 770-8503, Japan. [4]Tokushima University, 2-24 Shinkura-cho, Tokushima, 770-8501, Japan. *Correspondence and requests for materials should be addressed to A.Y. (yasuea@tokushima-u.ac.jp) or H.O. (hohuchi@okayama-u.ac.jp)

Supplementary Figure S1

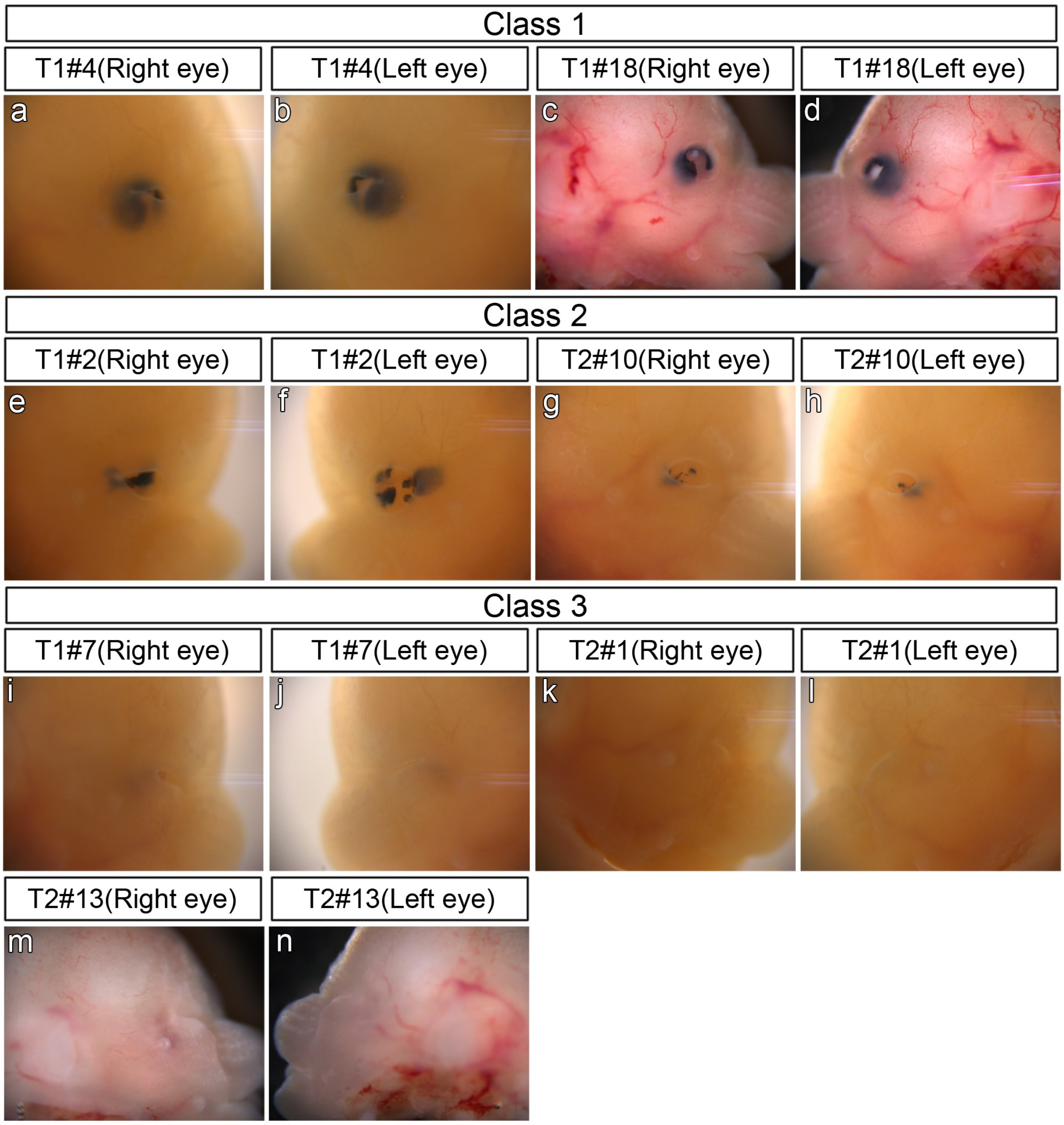

Supplementary Figure S1. Lateral views of the eye in the *Pax6*-CRISPR founder mice at E16.5.

Class 1 (a-d), class 2 (e-h), and class 3 (i-n).

Supplementary Figure S2

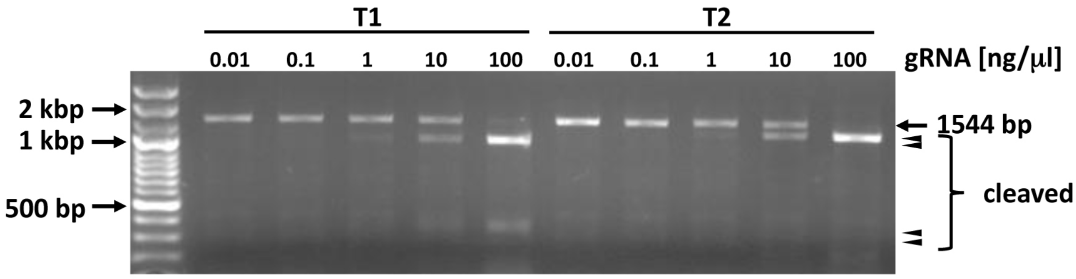

Supplementary Figure S2. Analysis of cleavage products by T1 or T2 sgRNA.

T1 and T2 sgRNAs were *in vitro* transcribed, respectively, and tested against its target sites in the mouse *Pax6* genomic region. A 1544 base pair-fragment of the *Pax6* genome was PCR-amplified using primers (5'-TTGTGGTCTTTTCTTCCTTCCT-3' and 5'-GGGCAAGGTAGACACACGAT-3'). The 1.5 kb fragment, T1 or T2 sgRNA, and recombinant Cas9 enzyme, using Guide-it sgRNA Screening Kit (Takara Bio Inc., Shiga, Japan), were combined in an *in vitro* cleavage reaction according to the manufacturer's protocol. Agarose gel analysis indicated that 100 ng of T1 or T2 sgRNA was each sufficient for 100% cleavage of the target fragment.

Supplementary Figure S3

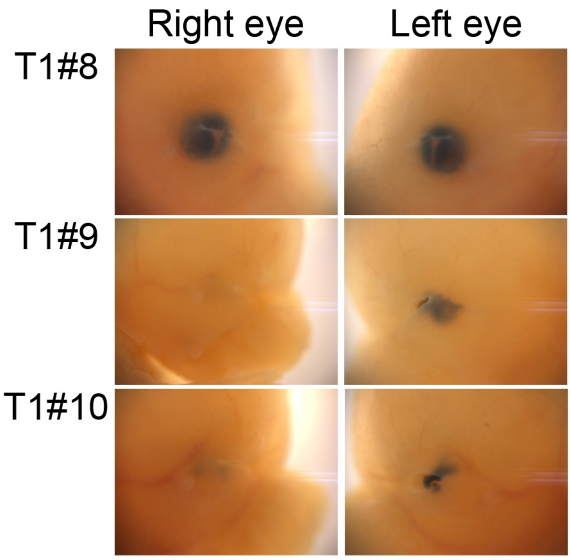

Supplementary Figure S3. Lateral views of the eyes.

In T1#8 embryo, both eyes exhibit class 1 phenotype. In T1#9 and T1#10 embryos, the right eyes exhibit a class 3 phenotype, while the left eyes exhibit a class 2 phenotype.

Supplementary Table S1. Test for association between genome-editing of the *Pax6* gene and occurrence of eye defects

				E11.5		E16.5	
Severity	Category	Observed	Expected	T1	T2	T1	T2
R=L	1	27	14.7	7	3	13	4
R>L	2	11	14.7	3	6	0	2
R<L	3	6	14.7	2	3	1	0
Total (n)		44		12	12	14	6

Statistical significance	
Chi-square	16.409
Degree of freedom	2
P	$P<0.0001$

Supplementary Table S2. Test for association between the parameters (right or left side) involved in occurrence of eye defects

				E11.5		E16.5	
Severity	Category	Observed	Expected	T1	T2	T1	T2
R>L	2	11	8.5	3	6	0	2
R<L	3	6	8.5	2	3	1	0
Total (n)		17		9	6	14	6

Statistical significance	
Chi-square	1.471
Degree of freedom	1
P	$P=0.225$

Another set of experiment (T1#XH and T2#XH series) was performed to examine whether there is a trend towards more severe eye defects in right vs left eyes examined at E11.5 or E16.5 (n=44). R>L, right eyes exhibit more severe defects than left eyes; R<L, left eyes exhibit more severe defects than right eyes; R=L, similar eye defects on both sides, by macroscopic observation. Table S1 shows a highly significant deviation among frequencies of R>L, R=L, and R<L. Table S2 shows no significant deviation between frequencies of R>L and R<L in occurrence of eye defects. These results indicate that R=L defects occur at a significantly higher frequency than defects of R>L or R<L.

Supplementary Figure S4

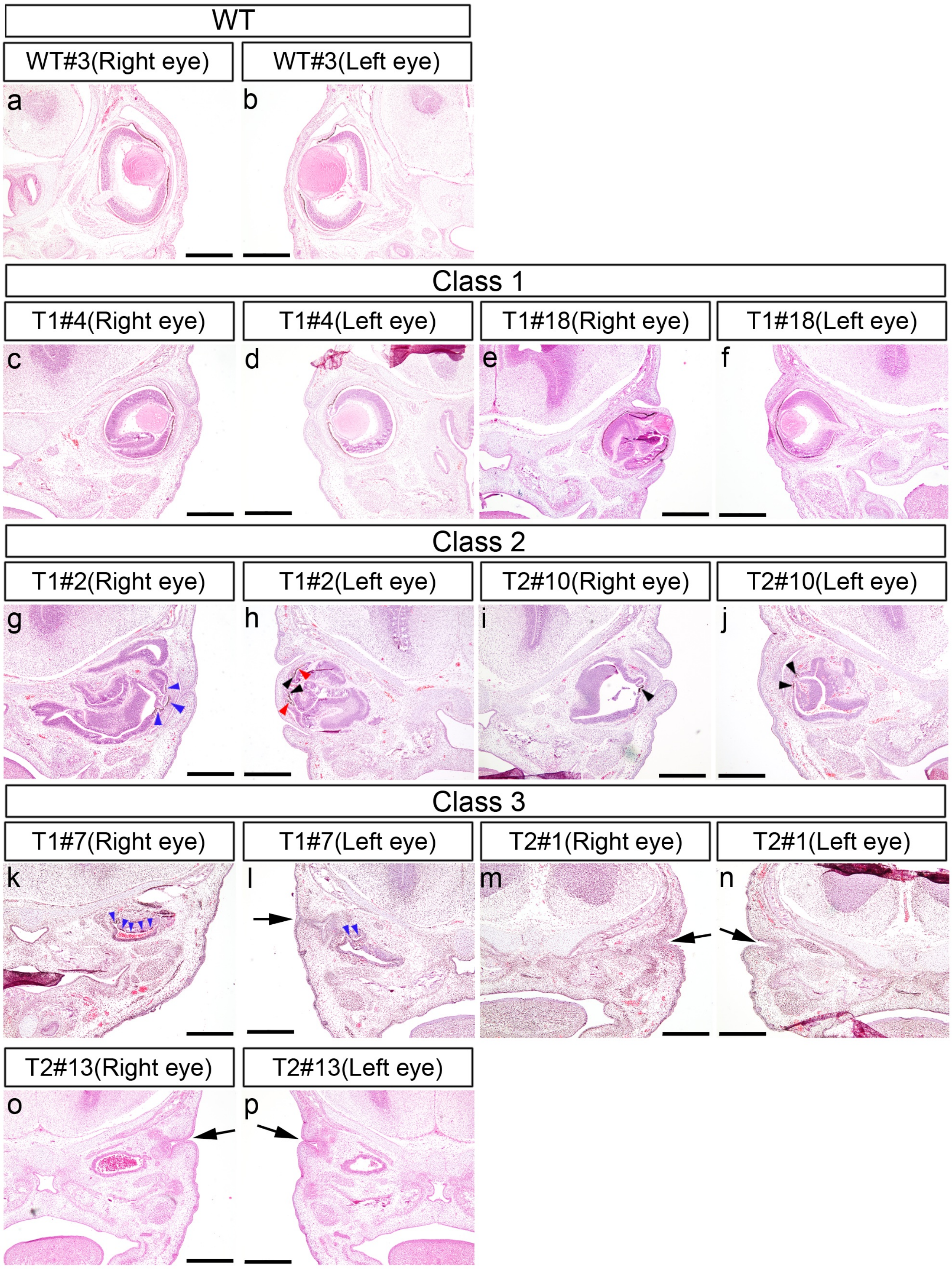

Supplementary Figure S4. Histology of the head region from the wild-type (a, b) and *Pax6*-CRISPR embryos (c-p) shown in Fig. 3.

HE-stained coronal sections. In (l-p), from the surface ectoderm, there was an invagination (arrow) that did not make contact with the pseudo-optic cup and no lens develops. Scale bars: 500 μm.

Supplementary Figure S5

```
                                                                     encoding truncated Pax6 proteins

a
        ProCysAspIleSerArgIleLeuGln
   WT   CCGTGCGACATTTCCCGAATTCTGCAGgtgatcctcccatggctgccctactcgccgctcc                    Phenotype
                                                                     (Total: 66)     Class 2
T1#2    CCGTGCGACATTTCCCGAATTCTGCAGgtgatcctcccatggctgccctactcgccgctcc    WT     (x14)
        CCGTGCGACATTTCCCGAATCACTGTGTGAGTGGGATTTCCAACTCTATGCCTGCAGgtga    -1+31  (x6)
        CCGTGCGACATTTCCCGAATCTCTGCAGgtgatcctcccatggctgccctactcgccgctcc   +1(C)  (x4)
        CCGTGCGACATTTCCCGAAT-CCTCCCAtgatcctcccatggctgccctactcgccgctcc    -8+7   (x2)
        CCGTGCGACATTTC--------TGCAGgtgatcctcccatggctgccctactcgccgctcc    -8     (x11)
        CCGTGCGACATTTCCCGA------------atcctcccatggctgccctactcgccgctcc    -12    (x8)
        CCGTGCGACATTTCCC-------------gatcctcccatggctgccctactcgccgctcc    -13    (x8)
        CCGTGCGACATTTCCCGAATTA------------------------ccctactcgccgctcc   -24+1  (x13)

                                                                     (Total: 52)     Class 1
T1#4    CCGTGCGACATTTCCCGAATTCTGCAGgtgatcctcccatggctgccctactcgccgctcc    WT     (x18)
        CCGTGCGACATTTCCCGAA-TCTGCAGgtgatcctcccatggctgccctactcgccgctcc    -1(T)  (x34)

                                                                     (Total: 79)     Class 2
T1#5H   CCGTGCGACATTTCCCGAATTCTGCAGgtgatcctcccatggctgccctactcgccgctcc    WT     (x5)
        CCGTGCGACATTTCCCGACATTTGCAGgtgatcctcccatggctgccctactcgccgctcc    -4+4   (x4)
        CCGTGCGACATTTCCCGAATTTCTGCAGgtgatcctcccatggctgccctactcgccgctcc   +1(T)  (x13)
        CCGTGCGACATTTC----ACTCTGCAGgtgatcctcccatggctgccctactcgccgctcc    -5+1   (x52)
        CCGTGCGACATTTC----------------------ccatggctgccctactcgccgctcc    -22    (x5)

                                                                     (Total: 52)     Class 1
T1#6    CCGTGCGACATTTCCCGAATTCTGCAGgtgatcctcccatggctgccctactcgccgctcc    WT     (x40)
        CCGTGCGACATTTCCCGAA---TGCAGgtgatcctcccatggctgccctactcgccgctcc    -3     (x11)
        CCGTGCGACATTTC----------------------ccatggctgccctactcgccgctcc    -22    (x1)

                                                                     (Total: 54)     Class 3
T1#7    CCGTGCGACATTTCC------CTGCAGgtgatcctcccatggctgccctactcgccgctcc    -6     (x23)
        CCGTGCGACATTTC-------------------ctcccatggctgccctactcgccgctcc    -19    (x31)

                                                                     (Total: 38)     Class 3
T1#11   CCGTGCGACATTTCCCGACAGGTGACATTTCTGCAGgtgatcctcccatggctgccctact    +9     (x16)
        CCGTGCGACATTTCCCGAA-TCTGCAGgtgatcctcccatggctgccctactcgccgctcc    -1(T)  (x22)

                                                                     (Total: 47)     Class 2
T1#12   CCGTGCGACATTTCCCGAATTCTGCAGgtgatcctcccatggctgccctactcgccgctcc    WT     (x21)
        CCGTGCGACATTTCCCGAATTTCTGCAggtgatcctcccatggctgccctactcgccgctc    +1(T)  (x1)
        CCGTGCGACATTTCCCGAA-TCTGCAGgtgatcctcccatggctgccctactcgccgctcc    -1(T)  (x19)
        CC-------------------------gtgatcctcccaaggctgccctactcgccgctcc    -25    (x6)

                                                                     (Total: 49)     Class 1
T1#18   CCGTGCGACATTTCCCGAATTCTGCAGgtgatcctcccatggctgccctactcgccgctcc    WT     (x14)
        CCGTGCGACATTTCCCGAATTTCTGCAGgtgatcctcccatggctgccctactcgccgctcc   +1(T)  (x2)
        CCGTGCGACATTTCCCGAA-TCTGCAGgtgatcctcccatggctgccctactcgccgctcc    -1(T)  (x2)
        CCGTGCGACATTTCC---------CAGgtgatcctcccatggctgccctactcgccgctcc    -9     (x20)
        CCGTGCGACATTTC----------------------ccatggctgccctactcgccgctcc    -22    (x11)


               ValSerAsnGlyCysValSerLysIleLeuGlyArgTyrTyrGluThrGlySer
   WT   aatgcagGTATCCAACGGTTGTGTGAGTAAAATTCTGGGCAGGTATTACGAGACTGGCTCC
                                                                     (Total: 30)     Class 3
T2#1    aatgcagGTATCCAACGGTTGTGTGAGTAAAATTCTGGGCAGGTATTACGAGACTGGCTCC    WT     (x1)
        aatgcagGTATCCAACGGTTGTGTGAGTAAAATTTCTGGGCAGGTATTACGAGACTGGCTCC   +1(T)  (x29)

                                                                     (Total: 89)     Class 3
T2#1H   aatgcagGTATCCAACGGTTGTGTGAGTAAAATTTCTGGGCAGGTATTACGAGACTGGCTCC   +1(T)  (x10)
        aatgcagGTATCCAACGGTTGTGTGAGTAAA-TTCTGGGCAGGTATTACGAGACTGGCTCC    -1(A)  (x37)
        aatgcagGTATCCAACGGTTGTGTGAGTAAAAT-CTGGGCAGGTATTACGAGACTGGCTCC    -1(T)  (x38)
        aatgcagGTATCCAACGGTTGTGTG------------GGCAGGTATTACGAGACTGGCTCC    -12    (x4)

                                                                     (Total: 32)     Class 3
T2#3    aatgcagGTATCCAACGGTTGTGTGAGTAAAATATCTGGGCAGGTATTACGAGACTGGCTCC   +1(A)  (x3)
        aatgcagGTATCCAACGGTTGTGTGAGTAAAATTTCTGGGCAGGTATTACGAGACTGGCTCC   +1(T)  (x3)
        aatgcagGTATCCAACGGTTGTGTGAGTAAAC-TCTGGGCAGGTATTACGAGACTGGCTCC    -2+1   (x14)
        aatgcagGTATCCAACGGTTGTGTGAGTAAAAT-CTGGGCAGGTATTACGAGACTGGCTCC    -1(T)  (x12)

                                                                     (Total: 46)     Class 3
T2#5H   aatgcagGTATCCAACGGTTGTGTGAGTAAAATTTCTGGGCAGGTATTACGAGACTGGCTCC   +1(T)  (x3)
        aatgcagGTATCCAACGGT---------------------AGGTATTACGAGACTGGCTCC    -21    (x4)
        aatgcagGTATCCAAC-------------------------GGTATTACGAGACTGGCTCC    -25    (x39)

                                                                     (Total: 40)     Class 2
T2#10   aatgcagGTATCCAACGGTTGTGTGAGTAAAATTCTGGGCAGGTATTACGAGACTGGCTCC    WT     (x13)
        aatgcagGTATCCAACGGTTGTGTGAGTAAAACAACCGTTGGGCAGGTATTACGAGACTGGCTCC  -3+7 (x5)
        aatgcagGTATCCAACGGTTGTGTGAGTAAAATAATCTGGGCAGGTATTACGAGACTGGCTCC  +2     (x16)
        aatgcagGTATCCAACGGTTGTGTGAGTAAAACTCTGGGCAGGTATTACGAGACTGGCTCC    -1+1   (x6)

                                                                     (Total: 31)     Class 3
T2#13   aatgcagGTATCCAACGGTTGTGTGAGTAAAA--CTGGGCAGGTATTACGAGACTGGCTCC    -2     (x23)
        aatgcagGTATCCAACGGTTGTGTGAG------------CAGGTATTACGAGACTGGCTCC    -12    (x3)
        aatgcagGTATCCAACGGTT----------------------GTATTACGAGACTGGCTCC    -22    (x5)
```

Supplementary Figure S5 (continued)

```
         AspIleSerArgIleLeuGln                   ValSerAsnGlyCysValSerLysIleLeuGlyArgTyr
WT       GACATTTCCCGAATTCTGCAGgtgatcctccca.........cagGTATCCAACGGTTGTGTGAGTAAAATTCTGGGCAGGTAT
                                                                                              Class 2
                                                                                                     (Total: 22)
T1+T2#6  GACATTTCCCGAATTCTGCAGgtgatcctccca.........cagGTATCCAACGGTTGTGTGAGTAAAATTCTGGGCAGGTAT  T1:WT,T2:WT      (x2)
         GACATTTCCCGAATTCTGCAGgtgatcctccca.........cagGTATCCAACGGTTGTGTGAGTAAAATTTCTGGGCAGGTAT T1:WT,T2:+1(T)(x2)
         GACATTTCCCGAAT-------gtgatcctccca.........cagGTATCCAACGGTTGTGTGAGTAAAATTTCTGGGCAGGTAT T1:-7,T2:+1(T)(x4)
         GACA--------------------------------------------------------------------GGTAT        -1064          (x13)
```

b

```
WT       MQNSHSGVNQLGGVFVNGRPLPDSTRQKIVELAHSGARPCDISRILQVSNGCVSKILGRYYETGSIRPRAIGGSKPRVATPEVVSKIAQY
         KRECPSIFAWEIRDRLLSEGVCTNDNIPSVSSINRVLRNLASEKQQMGADGMYDKLRMLNGQTGSWGTRPGWYPGTSVPGQPTQDGCQQQ
         EGGGENTNSISSNGEDSDEAQMRLQLKRKLQRNRTSFTQEQIEALEKEFERTHYPDVFARERLAAKIDLPEARIQVWFSNRRAKWRREEK
         LRNQRRQASNTPSHIPISSSFSTSVYQPIPQPTTPVSSFTSGSMLGRTDTALTNTYSALPPMPSFTMANNLPMQPPVPSQTSSYSCMLPT
         SPSVNGRSYDTYTPPHMQTHMNSQPMGTSGTTSTGLISPGVSVPVQVPGSEPDMSQYWPRLQ*

      T1
  -1+31  MQNSHSGVNQLGGVFVNGRPLPDSTRQKIVELAHSGARPCDISRITV*
     +9  MQNSHSGVNQLGGVFVNGRPLPDSTRQKIVELAHSGARPCDISRQVTFLQVSNGCVSKILGRYYETGSIRPRAIGGSKPRVATPE...RLQ*
+1(C/T)  MQNSHSGVNQLGGVFVNGRPLPDSTRQKIVELAHSGARPCDISRISAGIQRLCE*
   -4+4  MQNSHSGVNQLGGVFVNGRPLPDSTRQKIVELAHSGARPCDISRHLQVSNGCVSKILGRYYETGSIRPRAIGGSKPRVATPE......RLQ*
  -1(T)  MQNSHSGVNQLGGVFVNGRPLPDSTRQKIVELAHSGARPCDISRICRYPTVVS*
   -8+7  MQNSHSGVNQLGGVFVNGRPLPDSTRQKIVELAHSGARPCDISRILP*
     -3  MQNSHSGVNQLGGVFVNGRPLPDSTRQKIVELAHSGARPCDISR-MQVSNGCVSKILGRYYETGSIRPRAIGGSKPRVATPE......RLQ*
   -5+1  MQNSHSGVNQLGGVFVNGRPLPDSTRQKIVELAHSGARPCDISLCRYPTVV*
     -6  MQNSHSGVNQLGGVFVNGRPLPDSTRQKIVELAHSGARPCDIS--LQVSNGCVSKILGRYYETGSIRPRAIGGSKPRVATPE......RLQ*
     -8  MQNSHSGVNQLGGVFVNGRPLPDSTRQKIVELAHSGARPCDISAGIQRLCE*
     -9  MQNSHSGVNQLGGVFVNGRPLPDSTRQKIVELAHSGARPCDIS---QVSNGCVSKILGRYYETGSIRPRAIGGSKPRVATPE......RLQ*
    -12  MQNSHSGVNQLGGVFVNGRPLPDSTRQKIVELAHSGARPCDISRILPWLPYSPLPTHPHPAPSSCGPPL*
    -13  MQNSHSGVNQLGGVFVNGRPLPDSTRQKIVELAHSGARPCDISRSSHGCPTRRSLPTPTPPPRPAALHSKRPLFISYCKRY*
    -19  MQNSHSGVNQLGGVFVNGRPLPDSTRQKIVELAHSGARPCDISSHGCPTRRSLPTPTPPPRPAALHSKRPLFISYCKRY*
    -22  MQNSHSGVNQLGGVFVNGRPLPDSTRQKIVELAHSGARPCDISHGCPTRRSLPTPTPPPRPAALHSKRPLFISYCKRY*
  -24+1  MQNSHSGVNQLGGVFVNGRPLPDSTRQKIVELAHSGARPCDISRITLLAAPYPPPPRPLVLRPSTLSVLSSFLTVNATNY*
    -25  MQNSHSGVNQLGGVFVNGRPLPDSTRQKIVELAHSGARP*

      T2
   -3+7  MQNSHSGVNQLGGVFVNGRPLPDSTRQKIVELAHSGARPCDISRILQVSNGCVSKTTVGQVLRDWLHQTQGNRRE*
     +2  MQNSHSGVNQLGGVFVNGRPLPDSTRQKIVELAHSGARPCDISRILQVSNGCVSKIIWAGITRLAPSDPGQSEGVSQEWRLQKL*
+1(A/T)  MQNSHSGVNQLGGVFVNGRPLPDSTRQKIVELAHSGARPCDISRILQVSNGCVSKISGQVLRDWLHQTQGNRRE*
   -1+1  MQNSHSGVNQLGGVFVNGRPLPDSTRQKIVELAHSGARPCDISRILQVSNGCVSKTLGRYYETGSIRPRAIGGSKPRVATPE......RLQ*
  -1(A)  MQNSHSGVNQLGGVFVNGRPLPDSTRQKIVELAHSGARPCDISRILQVSNGCVSKFWAGITRLAPSDPGQSEGVSQEWRLQKL*
  -1(T)  MQNSHSGVNQLGGVFVNGRPLPDSTRQKIVELAHSGARPCDISRILQVSNGCVSKIWAGITRLAPSDPGQSEGVSQEWRLQKL*
   -2+1  MQNSHSGVNQLGGVFVNGRPLPDSTRQKIVELAHSGARPCDISRILQVSNGCVSKLWAGITRLAPSDPGQSEGVSQEWRLQKL*
     -2  MQNSHSGVNQLGGVFVNGRPLPDSTRQKIVELAHSGARPCDISRILQVSNGCVSNGCVSKTGQVLRDWLHQTQGNRRE*
    -12  MQNSHSGVNQLGGVFVNGRPLPDSTRQKIVELAHSGARPCDISRILQVSNGCVS----RYYETGSIRPRAIGGSKPRVATPE......RLQ*
    -21  MQNSHSGVNQLGGVFVNGRPLPDSTRQKIVELAHSGARPCDISRILQVSNG-------RYYETGSIRPRAIGGSKPRVATPE......RLQ*
    -22  MQNSHSGVNQLGGVFVNGRPLPDSTRQKIVELAHSGARPCDISRILQVSNGCITRLAPSDPGQSEGVSQEWRLQKL*
    -25  MQNSHSGVNQLGGVFVNGRPLPDSTRQKIVELAHSGARPCDISRILQVSNGITRLAPSDPGQSEGVSQEWRLQKL*

         T1+T2
T1:WT,T2:+1(T)  MQNSHSGVNQLGGVFVNGRPLPDSTRQKIVELAHSGARPCDISRILQVSNGCVSKISGQVLRDWLHQTQGNRRE*
T1:-7,T2:+1(T)  MQNSHSGVNQLGGVFVNGRPLPDSTRQKIVELAHSGARPCDISRMYPTVV*
         -1064  MQNSHSGVNQLGGVFVNGRPLPDSTRQKIVELAHSGARPCD----------------RYYETGSIRPRAIGGSKPRVATPE......RLQ*
```

Sequencing primers for T1+T2 were 5’-TTGTGGTCTTTTCTTCCTTCCT-3’ and 5’-GGGCAAGGTAGACACACGAT-3’. T1#5H, T2#1H, and T2#5H embryos were from another set of experiments, which is different from those shown in Tables 1 and 2.

Supplementary Table S3. Summary of genotypes for the HE-stained embryos (E16.5) shown in Fig. 3 and Supplementary Fig. S4

Phenotype Class (Microscopic)	1	1	2	2	3	3	3
Embryo No.	T1#4	T1#18	T1#2	T2#10	T1#7	T2#1	T2#13
Pax6 Mutation: Truncation	34 (65.4%)	15(30.6%)	44 (66.7%)	21(52.5%)	31(57.4%)	29(96.7%)	28(90.3%)
Pax6 Mutation: In-frame (In/Del/Sub)	0	20(40.8%;3aaDel)	8(12.1%;4aaDel)	6(15%;1aaSub)	23(42.6%;2aaDel)	0	3(9.7%;4aaDel)
Wild-type (%)	18 (34.6%)	14(28.6%)	14(21.2%)	13(32.5%)	0	1(3.3%)	0
Sequencing Clone No.	52	49	66	40	54	30	31
Mosaicism	2	5	8	4	2	2	3

1aaSub:1 amino acid substitution

Supplementary Table S4. Summary of genotypes for the immunostained embryos (E16.5) shown in Fig. 4

Phenotype Class (Microscopic)	1	2	3	3
Embryo No.	T1#6	T1#12	T1#11	T2#3
Pax6 Mutation: Truncation	1(1.9%)	26 (54.3%)	22 (57.9%)	32(100%)
Pax6 Mutation: In-frame (In/Del)	11(21.2%; 1aaDel)	0	16 (42.1%; 3aaIn)	0
Wild-type (%)	40 (76.9%)	21 (45.7%)	0	0
Sequencing Clone No.	52	47	38	32
Mosaicism	3	4	2	4

1aaDel: 3 amino acid deletion 3aaIn: 3 amino acid insertion

Supplementary Table S5. Data for Fig. 4v

Embryo No.	Class	X axis	Y axis
		% Pax6 truncated mutations	% Pax6 (+) area
WT#4		0	59.5
WT#5		0	53.2
T1#6	1	1.9	86.4
T1+T2 #6*	2	27.3	45.3
T1#12	2	54.3	75.1
T1#11**	3	57.9	77.6
T1#5H	2	88.6	44.6
T2#5H	3	91.3	36
T2#1H	3	95.5	46.3
T2#3	3	100	16.1

*also had an in-frame mutation of 17 amino acid deletion in the PD (59.0%)

**also had an in-frame mutation of 3 amino acid insertion in the PD (42.1%)

Supplementary Table S6. Data for Fig. 4w

Embryo No.	Class	X axis	Y axis
		Pax6 (+) area (μm^2)	Tuj1 (+) area (μm^2)
T2#3	3	6143	1578
T2#1H	3	10629	345
T2#5H	3	23409	10446
T1#11*	3	61027	24776
WT#5		115027	144856
T1#5H	2	116751	90795
T1+T2 #6**	2	142972	134391
WT#4		156576	193865
T1#6	1	219048	57744
T1#12	2	547239	231312

*also had an in-frame mutation of 3 amino acid insertion in the PD domain (42.1%)

**also had an in-frame mutation of 17 amino acid deletion in the PD domain (59.0%)

Supplementary Figure S6

encoding truncated Pax6 proteins

a

```
          ProCysAspIleSerArgIleLeuGln
WT        CCGTGCGACATTTCCCGAATTCTGCAGgtgatcctcccatggctgccctactcgccgctcc         Phenotype(R-L)
                                                                    (Total: 57)   Class 2-3
T1#2_e11  CCGTGCGACATTTCCCGAATTCTGCAGgtgatcctcccatggctgccctactcgccgctcc          WT     (x10)
          CCGTGCGACATTTCCCGAATTTTCTGCAGgtgatcctcccatggctgccctactcgccgctcc        +2     (x12)
          CCGTGCGACATTTCCCGAATTTCTGCAGgtgatcctcccatggctgccctactcgccgctcc         +1(T)  (x4)
          CCGTGCGACATTTCCCGAATG-----Ggtgatcctcccatggctgccctactcgccgctcc          -6+1   (x12)
          CCGTGCGACATTTC--------TGCAGgtgatcctcccatggctgccctactcgccgctcc          -8     (x19)

                                                                    (Total: 28)   Class 3
T1#9_e11  CCGTGCGACATTTCCC--AT-------gtgatcctcccatggctgccctactcgccgctcc          -9     (x8)
          CCGTGCGACATTTC----------------------ccatggctgccctactcgccgctcc          -22    (x20)


                 ValSerAsnGlyCysValSerLysIleLeuGlyArgTyrTyrGluThrGlySer
WT        aatgcagGTATCCAACGGTTGTGTGAGTAAAATTCTGGGCAGGTATTACGAGACTGGCTCC
                                                                    (Total: 56)   Class 2/3-2/3
T2#2_e11  aatgcagGTATCCAACGGTTGTGTGAGTAAAATTTCTGGGCAGGTATTACGAGACTGGCTCC         +1(T)  (x5)
          aatgcagGTATCCAACGGTTGTGTGAGTAA---CCTGGGCAGGTATTACGAGACTGGCTCC          -4+1   (x13)
          aatgcagGTATCCAACGGT-----------AATACTGGGCAGGTATTACGAGACTGGCTCC          -12+1  (x15)
          aatgcagGTATCCAACGGTTGTG--------------GGCAGGTATTACGAGACTGGCTCC          -14    (x8)
          aatgcagGTATCCAAC-------------------------GGTATTACGAGACTGGCTCC          -25    (x15)

                                                                    (Total: 47)   Class 3-3
T2#4-e11  aatgcagGTATCCAACGGTTGTGTGAGTAAAATTTCTGGGCAGGTATTACGAGACTGGCTCC         +1(T)  (x19)
          aatgcagGTATCCAACGGTTGTGTGAGTAAAAT-CTGGGCAGGTATTACGAGACTGGCTCC          -1(T)  (x10)
          aatgcagGTATCCAACGGTTGTGTGAGTAAA-TTCTGGGCAGGTATTACGAGACTGGCTCC          -1(A)  (x18)

                                                                    (Total: 24)   Class 2/3-2
T2#7_e11  aatgcagGTATCCAACGGTTGTGTGAGTAAAATGTCTGGGCAGGTATTACGAGACTGGCTCC         +1(G)  (x6)
          aatgcagGTATCCAACGGTTGTGTGAG-----------GCAGGTATTACGAGACTGGCTCC          -11    (x3)
          aatgcagGTATCCAACGGTTGTGTG------------GGCAGGTATTACGAGACTGGCTCC          -12    (x15)
```

b

```
WT      MQNSHSGVNQLGGVFVNGRPLPDSTRQKIVELAHSGARPCDISRILQVSNGCVSKILGRYYETGSIRPRAIGGSKPRVATPEVVSKIAQY
        KRECPSIFAWEIRDRLLSEGVCTNDNIPSVSSINRVLRNLASEKQQMGADGMYDKLRMLNGQTGSWGTRPGWYPGTSVPGQPTQDGCQQQ
        EGGGENTNSISSNGEDSDEAQMRLQLKRKLQRNRTSFTQEQIEALEKEFERTHYPDVFARERLAAKIDLPEARIQVWFSNRRAKWRREEK
        LRNQRRQASNTPSHIPISSSFSTSVYQPIPQPTTPVSSFTSGSMLGRTDTALTNTYSALPPMPSFTMANNLPMQPPVPSQTSSYSCMLPT
        SPSVNGRSYDTYTPPHMQTHMNSQPMGTSGTTSTGLISPGVSVPVQVPGSEPDMSQYWPRLQ*

T1
+2      MQNSHSGVNQLGGVFVNGRPLPDSTRQKIVELAHSGARPCDISRIFCR*
+1(T)   MQNSHSGVNQLGGVFVNGRPLPDSTRQKIVELAHSGARPCDISRISAGIQRLCE*
-6+1    MQNSHSGVNQLGGVFVNGRPLPDSTRQKIVELAHSGARPCDISRMGIQRLCE*
-8      MQNSHSGVNQLGGVFVNGRPLPDSTRQKIVELAHSGARPCDISAGIQRLCE*
-9      MQNSHSGVNQLGGVFVNGRPLPDSTRQKIVELAHSGARPCDISH---VSNGCVSKILGRYYETGSIRPRAIGGSKPRVATPE......RLQ*
-22     MQNSHSGVNQLGGVFVNGRPLPDSTRQKIVELAHSGARPCDISHGCPTRRSLPTPTPPPRPAALHSKRPLFISYCKRY*

T2
+1(G)   MQNSHSGVNQLGGVFVNGRPLPDSTRQKIVELAHSGARPCDISRILQVSNGCVSKMSGQVLRDWLHQTQGNRRE*
+1(T)   MQNSHSGVNQLGGVFVNGRPLPDSTRQKIVELAHSGARPCDISRILQVSNGCVSKISGQVLRDWLHQTQGNRRE*
-1(A)   MQNSHSGVNQLGGVFVNGRPLPDSTRQKIVELAHSGARPCDISRILQVSNGCVSKFWAGITRLAPSDPGQSEGVSQEWRLQKL*
-1(T)   MQNSHSGVNQLGGVFVNGRPLPDSTRQKIVELAHSGARPCDISRILQVSNGCVSKIWAGITRLAPSDPGQSEGVSQEWRLQKL*
-4+1    MQNSHSGVNQLGGVFVNGRPLPDSTRQKIVELAHSGARPCDISRILQVSNGCVSKI-GRYYETGSIRPRAIGGSKPRVATPE......RLQ*
-11     MQNSHSGVNQLGGVFVNGRPLPDSTRQKIVELAHSGARPCDISRILQVSNGCVRQVLRDWLHQTQGNRRE*
-12+1   MQNSHSGVNQLGGVFVNGRPLPDSTRQKIVELAHSGARPCDISRILQVSNGNTGQVLRDWLHQTQGNRRE*
-12     MQNSHSGVNQLGGVFVNGRPLPDSTRQKIVELAHSGARPCDISRILQVSNGCV----GRYYETGSIRPRAIGGSKPRVATPE......RLQ*
-14     MQNSHSGVNQLGGVFVNGRPLPDSTRQKIVELAHSGARPCDISRILQVSNGCGQVLRDWLHQTQGNRRE*
-25     MQNSHSGVNQLGGVFVNGRPLPDSTRQKIVELAHSGARPCDISRILQVSNGITRLAPSDPGQSEGVSQEWRLQKL*
```

Supplementary Figure S7

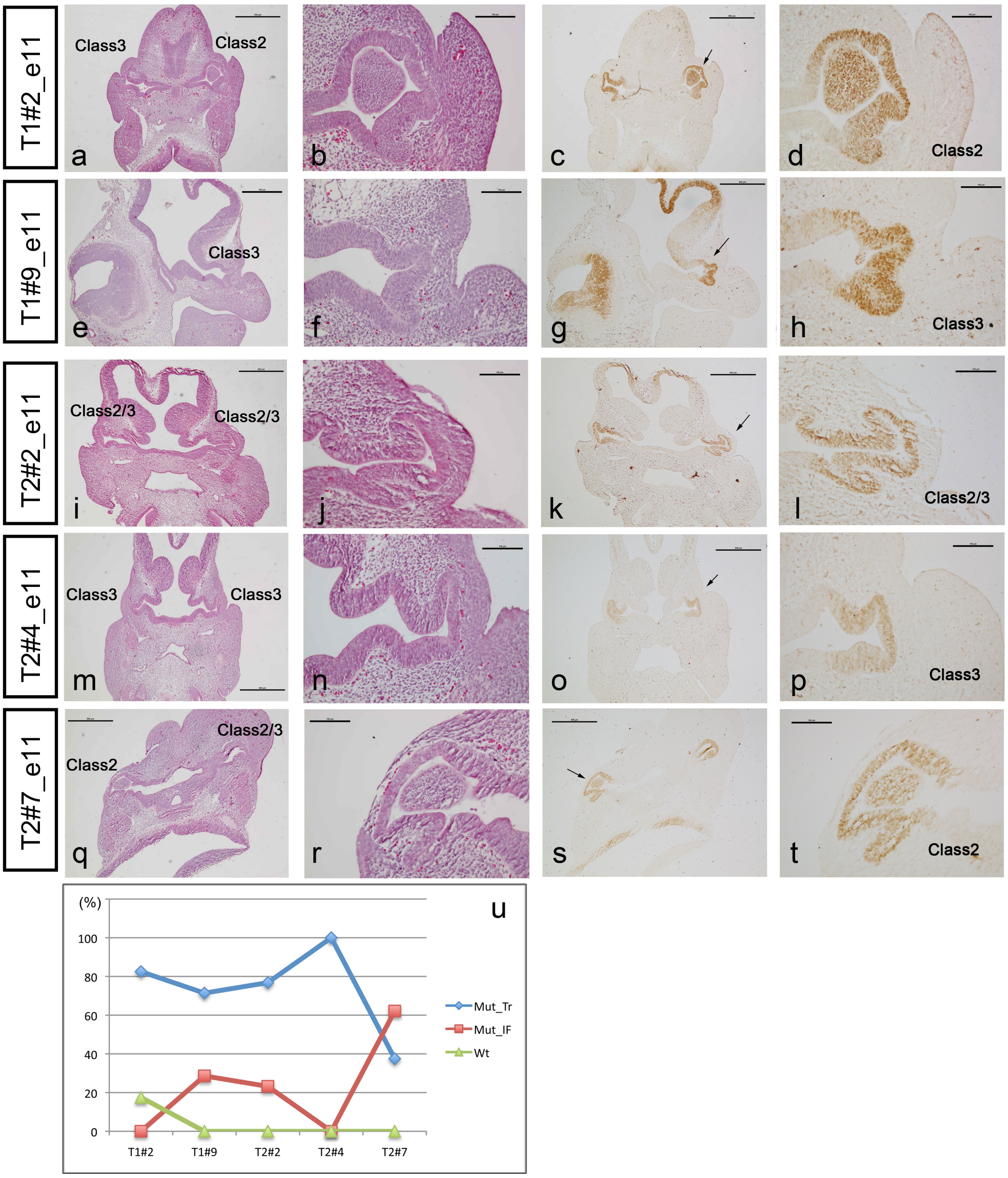

Supplementary Figure S7. HE-stained and immunostained head tissues from founder mice at E11.5.

Transverse sections are shown. Brown staining in (c, d, g, h, k, l, o, p, s, t) indicates localization of Pax6 proteins, wild-type and mutated. (u) Summary of genotypes for each embryo. Details are shown in Supplementary Fig. S6 and Table S5. Scale bars: 500 μm in (a, c, e, g, i, k, m, o, q, s); 100 μm in (b, d, f, h, j, l, n, p, r, t).

Supplementary Table S7. Summary of genotypes for the HE-stained embryos (E11.5) shown in Figure S6

Phenotype Class (Microscopic) R/L	Class3/Class2	n.d./Class3	Class2/3	Class3	Class2/Class2/3
Embryo No.	T1#2_e11	T1#9_e11	T2#2_e11	T2#4_e11	T2#7_e11
Pax6 Mutation: Truncation	47(82.5%)	20(71.4%)	43(76.8%)	47(100%)	9(37.5%)
Pax6 Mutation: In-frame (In/Del)	0	8(28.6%; 3aaDel)	13(23.2%; 1aaDel)	0	15(62.5%)
Wild-type (%)	10(17.5%)	0	0	0	0
Sequencing Clone No.	57	28	56	47	24
Mosaicism	5	2	5	3	3

n.d.: The optic primordium could not be determined on the sections.

Supplementary Figure S8

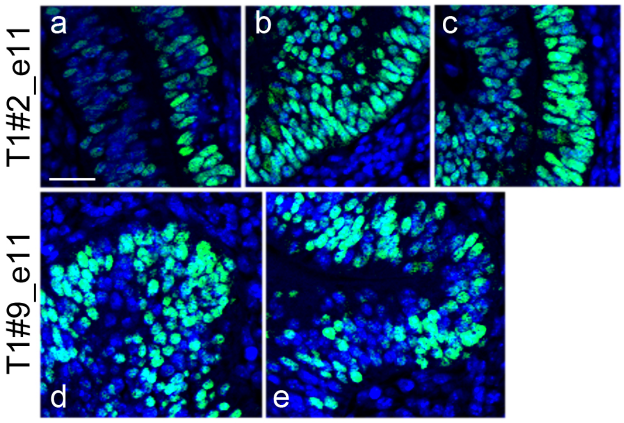

Supplementary Figure S8. Pax6 immunoreactivity in E11.5 *Pax6*-mosaic optic vesicle.

High magnification images of optic vesicles shown in Fig. 5a (a-c) and Fig. 5c (d, e). Scale bar: 25 μm.
