# Supplementary material for: Relationship between somatic mosaicism of Pax6 mutation and variable developmental eye abnormalities—an analysis of CRISPR genome-edited mouse embryos
Source: Sci Rep. 2017 Mar 3;7:53. doi: 10.1038/s41598-017-00088-w (PMC5428340; doi:10.1038/s41598-017-00088-w)
Supplement: Supplementary file 2 — Supplementary Information 2 [file 41598_2017_88_MOESM2_ESM.pdf]

Supplementary Information 2

Relationship between somatic mosaicism of *Pax6* mutation and variable developmental eye abnormalities—an analysis of CRISPR genome-edited mouse embryos

Akihiro Yasue[1,*], Hitomi Kono[2], Munenori Habuta[2], Tetsuya Bando[2], Keita Sato[2], Junji Inoue[2], Seiichi Oyadomari[3], Sumihare Noji[4], Eiji Tanaka[1] and Hideyo Ohuchi[2,*]

[1]Department of Orthodontics Dentofacial Orthopedics, Institute of Biomedical Sciences, Tokushima University Graduate School, 3-18-15 Kuramoto-cho, Tokushima, 770-8504, Japan. [2]Department of Cytology and Histology, Okayama University Graduate School of Medicine, Dentistry and Pharmaceutical Sciences, 2-5-1 Shikata-cho, Kita-ku, Okayama, 700-8558, Japan. [3]Division of Molecular Biology, Institute for Advanced Enzyme Research, Tokushima University, 3-18-15 Kuramoto-cho, Tokushima, 770-8503, Japan. [4]Tokushima University, 2-24 Shinkura-cho, Tokushima, 770-8501, Japan. *Correspondence and requests for materials should be addressed to A.Y. (yasuea@tokushima-u.ac.jp) or H.O. (hohuchi@okayama-u.ac.jp)

Supplementary Table S8. List of off-target sites located in exonic regions

**T1**	GGAGGATCACCTGCAGAATT	CGG				
OT1	CGAGGCTCACCTGCAGGATG	AGG	0.2	4	chr1:-36556408	cyclin M4 (Cnnm4)
OT2	GGAGGATCACCTTCGGAACC	GGG	0	4	chr1:-75249699	protein tyrosine phosphatase, receptor type, N (Ptprn)
OT3	AGAGGATCCTCTGCAGAATG	CAG	0.4	4	chr1:+155584967	RIKEN cDNA C230024C17 gene (C230024C17Rik), long non-coding RNA
OT4	GGAGGAACAACAGCAGAATA	AGG	0.2	4	chr10:+121005198	TANK-binding kinase 1 (Tbk1)
OT5	GGATCATCACATGGAGAATT	GAG	0.1	4	chr10:+126440482	advillin (Avil)
OT6	GCAGCATCACCTACAGCATT	GAG	0.2	4	chr11:-55109664	FAT atypical cadherin 2 (Fat2)
OT7	GGAAGCTCACCTGCAGCATG	GAG	0.2	4	chr11:-79498870	RAB11 family interacting protein 4 (class II) (Rab11fip4)
OT8	GGAGGATCACCTGCAGCGGA	TGG	0	4	chr11:+118344242	endo-beta-N-acetylglucosaminidase (Engase)
OT9	GGAGACTCACCTGCAATATT	TGG	0.1	4	chr12:-57591978	RIKEN cDNA E030019B13 gene (E030019B13Rik), long non-coding RNA
OT10	GTATGAGCACCTGCAGAAGT	GGG	0.4	4	chr12:-113104690	protein phosphatase 1, regulatory (inhibitor) subunit 13B (Ppp1r13b)
OT11	CTCGGATCACCTGCAGCATT	GGG	0.6	4	chr15:+82990588	cytochrome b5 reductase 3 (Cyb5r3)
OT12	GGAGGACCACCTCTAGAAAT	TAG	0	4	chr15:+89119580	SET binding factor 1 (Sbf1)
OT13	AGAGTATCATCTTCAGAATT	CAG	0.5	4	chr18:-60754220	synaptopodin (Synpo)
OT14	GGAGGATCCCCTGCAGTGTT	AAG	0.1	3	chr2:-157021534	retinoblastoma-like 1 (p107) (Rbl1)
OT15	GAAGGCTCAGCTGCAGAATG	AGG	0.4	4	chr4:+115131400	cytochrome P450, family 4, subfamily a, polypeptide 30b (Cyp4a30b)
OT16	GGATCATCACCTGCAGAATC	GAG	1.4	3	chr5:+69969380	glucosamine-6-phosphate deaminase 2 (Gnpda2)
OT17	GGGTGGTCACCTGCAGAATT	AGG	1.4	3	chr6:+72582151	transcription factor 7 like 1 (T cell specific, HMG box) (Tcf7l1)
OT18	GAAGGAACACCTGCAGAATA	AAG	1	3	chr7:-20613786	NLR family, pyrin domain containing 9B (Nlrp9b)
OT19	GAAGGAACACCTGCAGGATA	AAG	0.2	4	chr7:+27165589	NLR family, pyrin domain containing 9C (Nlrp9c)
OT20	GAAGGAACACCTGCAGGATA	AAG	0.2	4	chr7:-27347447	NLR family, pyrin domain containing 9A (Nlrp9a)
**T2**	GGTTGTGTGAGTAAAATTCT	GGG				
OT1	GCATGTGTGAATTAAATTCT	TAG	0.3	4	chr1:-14871933	transient receptor potential cation channel, subfamily A, member 1 (Trpa1)
OT2	GGTTGTTTAGGTAAAATTAT	AGG	0.2	4	chr1:-58580968	family with sequence similarity 126, member B (Fam126b),
OT3	CTTTGTGTCAGTAAAGTTCT	TGG	0.2	4	chr10:+82951250	aldehyde dehydrogenase 1 family, member L2 (Aldh1l2)
OT4	GGAGGTGTGTGTTAAATTCT	AGG	0.5	4	chr11:-69171183	chromodomain helicase DNA binding protein 3 (Chd3)
OT5	GTTTTTCTGAATAAAATTCT	TGG	0.5	4	chr11:-77505213	nuclear fragile X mental retardation protein interacting protein 2 (Nufip2)
OT6	GGTTGTGTGAGAAAAATCTA	TGG	0	4	chr14:-65212409	Mir124-1 host gene (non-protein coding) (Mir124a-1hg)
OT7	GATTGTGTGAGTAAATTTTT	AAG	0.2	3	chr15:-36530293	poly(A) binding protein, cytoplasmic 1 (Pabpc1)
OT8	GTTTGTTTGAGTAACATTGT	TGG	0.1	4	chr17:-55764005	vomeronasal 2, receptor 118 (Vmn2r118),
OT9	GGTTCTGTGAGTAAAACCCA	GGG	0	4	chr19:-41726099	slit homolog 1 (Drosophila) (Slit1)
OT10	GGCTGTGTCAGCAAAATCCT	GGG	0.1	4	chr19:+44835506	paired box 2 (Pax2)
OT11	GTTTCTGTTAGTAAAATTTT	TGG	0.3	4	chr2:-37288374	zinc finger and BTB domain containing 26 (Zbtb26)
OT12	GGTTGCTTAAGTAAACTTCT	TAG	0.1	4	chr2:+120686417	ubiquitin protein ligase E3 component n-recognin 1 (Ubr1)
OT13	GGATGTCTGAGGAAGATTCT	GAG	0.1	4	chr2:-180969533	helicase with zinc finger 2, transcriptional coactivator (Helz2)
OT14	GCTTCTGTAAATAAAATTCT	GGG	0.5	4	chr3:-98102948	3-hydroxy-3-methylglutaryl-Coenzyme A synthase 2 (Hmgcs2)
OT15	GGTTGTGTCAGCAAAATTCT	TGG	1.7	2	chr4:-44710392	paired box 5 (Pax5)
OT16	GGCTGTGTGAGCAAGATCCT	AGG	0	4	chr6:-28396312	paired box 4 (Pax4), transcript variant 3

Predicted each for T1 and T2 gRNAs using the Zhang laboratory CRISPR design tool. Red bases indicate sequence differences from target sequence.
